# Enhancing Childhood-Educator Knowledge and Confidence: Virtual “Coffee Chat” Interventions with Medical Professionals

**DOI:** 10.7759/cureus.87290

**Published:** 2025-07-04

**Authors:** Prutha Patel, Yash Desai, Harsha Bhagtani, David Redden, Sofia Abraham-Hardee

**Affiliations:** 1 Pediatrics, Edward Via College of Osteopathic Medicine, Blacksburg, USA; 2 Research and Biostats, Edward Via College of Osteopathic Medicine, Auburn, USA

**Keywords:** community health education, confidence and knowledge outcomes, early childhood education, educator professional development, health education, integrated management of childhood illness, interdisciplinary collaboration, pediatric health literacy, public health intervention, virtual learning

## Abstract

Context

Early childhood educators (ECEs) are central to the development and care of children, but they often lack adequate access to resources and expert guidance for managing common childhood health issues. These challenges can lead to significant stress for both educators and parents. To address this gap, a pilot intervention involving virtual “coffee chat” sessions was implemented. These sessions aimed to provide ECEs with evidence-based knowledge and practical strategies to handle frequent childhood health concerns.

Objective

The primary objective of this study was to enhance the knowledge and confidence of ECEs in addressing common health and behavioral issues among children in the New River Valley, USA. The sessions, facilitated by pediatricians and medical students, covered a range of topics identified as high-priority by educators. The goal was to create a sustainable educational model for improving child health management in childcare settings.

Methods

This study used a self-designed pre- and post-survey format to measure the effectiveness of eight virtual “coffee chat” sessions conducted over two years. The initial sessions focused on educators in the New River Valley, USA, and the final session expanded across Virginia, USA. Topics included common illnesses (e.g., cough, diarrhea), trauma management, obesity, and behavioral issues such as self-regulation and temper tantrums. Each session consisted of a brief evidence-based presentation followed by interactive discussions. Participants were recruited through local and statewide child care organizations, and surveys were used to assess changes in their confidence and knowledge levels. Descriptive statistics and a Wilcoxon Signed Rank test were applied to analyze the data, while qualitative feedback from open-ended responses was categorized thematically.

Results

Across the eight sessions, 30 registrations were recorded, with 19 participants, some of whom attended multiple sessions. Pre- and post-survey results demonstrated significant improvements in knowledge and confidence, with an overall p-value of <0.001. Six out of eight topics showed increased post-survey scores, with the session on self-regulation and temper tantrums, which had the largest cohort (N=10), yielding a significant improvement (p=0.0041), highlighting its broad impact and relevance. Feedback from participants highlighted the value of practical, evidence-based strategies and the opportunity to engage directly with healthcare professionals. However, variability was observed in some topics, such as diarrhea, where post-survey scores declined slightly, pointing to potential areas for improvement.

Conclusion

Virtual “coffee chat” sessions proved to be an effective, scalable method for improving the health management skills of ECEs. The intervention’s interactive and accessible format was particularly appreciated by participants and shows promise as a model for ongoing professional development. However, given the study's design and sample size, these findings should be interpreted as preliminary. Future efforts should focus on refining content, expanding participation, and exploring long-term impacts on both educator practices and child outcomes.

## Introduction

Early childhood educators (ECEs) play a crucial role in the health and well-being of children, particularly in childcare settings where the risk of illness, infection, and injury is high. Common concerns such as diarrhea, cough, trauma, and other childhood health issues can disrupt classroom dynamics and cause distress for both educators and parents. Despite the importance of managing these concerns effectively, many educators lack sufficient training or direct access to healthcare professionals, leaving them underprepared to make informed decisions about a child's health in real-time. While resources and professional development opportunities exist, they are often limited in scope, accessibility, or relevance to the specific challenges faced by ECEs. Many educators express the need for practical, evidence-based guidance that addresses their day-to-day experiences while also equipping them to communicate effectively with parents about health concerns.

The need for improved training in child health management for ECEs has been emphasized in the literature. For instance, Peetoom et al. demonstrated that tailored interventions could enhance childcare providers’ decision-making and confidence in handling common health issues, such as fever and infections, through cluster-randomized trials [1]. However, much of the existing research focuses on isolated health issues rather than providing a comprehensive approach to the common challenges faced by educators.

Similarly, studies have identified gaps in knowledge and attitudes among childcare providers. Research conducted in Shanghai found that preschool staff lacked confidence and adequate knowledge in pediatric first aid and managing common illnesses, which could delay effective care [2]. Another study by Juhn et al. highlighted that many child care program directors were underprepared to manage chronic conditions like asthma, further emphasizing the need for ongoing education tailored to their specific professional environment [3].

While professional development programs have shown positive impacts on child outcomes, as noted in a meta-analysis by Brunsek et al., most of these interventions focus on broader developmental outcomes rather than health-related training [4]. Similar studies have reinforced the importance of targeted professional development for early educators, though health-specific topics remain underrepresented [5-12]. These findings highlight the need for comprehensive, accessible, and practical health-related training programs.

Given the constraints faced by educators, virtual interventions offer a promising solution. Studies have highlighted the importance of flexible education formats, particularly in underserved regions, to bridge the gap in professional development opportunities [13]. Virtual formats like the “coffee chat” sessions described in this study allow educators to engage directly with healthcare professionals, offering evidence-based insights and real-time discussions tailored to their needs. In this context, “coffee chats” refer to informal yet structured virtual meetings where ECEs can ask questions, share concerns, and learn from pediatricians and medical students in a conversational setting.

To address these gaps, this study piloted an innovative educational intervention known as “coffee chat” sessions. These virtual discussions brought together ECEs, pediatricians, and medical students to explore common childhood health topics such as diarrhea, cough, trauma, fever, rash, obesity, self-regulation and temper tantrums, and social and separation anxiety. The sessions aimed to enhance educators' confidence and knowledge in managing these issues while fostering open dialogue and collaboration between educators and healthcare experts. By tailoring the content to educators' needs and offering a flexible virtual format, this intervention seeks to provide a sustainable model for professional development in childcare settings while addressing the identified gaps in health-related training for ECEs.

This article was previously presented as a poster at the Edward Via College of Osteopathic Medicine - Virginia (VCOM-VA) Research Day in 2025.

Objective

The goal of this pilot project was to improve the knowledge and confidence of ECEs in managing common childhood illnesses. The virtual “coffee chat” interventions, conducted via Zoom, initially focused on educators in the New River Valley, USA, but later expanded to include participants from across Virginia, USA, for the final sessions. These sessions provided expert guidance on healthcare topics tailored to the needs of ECEs. The primary objectives were to offer evidence-based information and foster an open dialogue between ECEs and healthcare professionals. This project seeks to create a sustainable model for ongoing education and support in childcare settings.

## Materials and methods

This study involved the use of human participants and was reviewed by the Edward Via College of Osteopathic Medicine Institutional Review Board (IRB). The IRB determined that the project does not meet the definition of human subject research under the purview of the IRB according to federal regulations (Approval #: 2120724-1).

Study design

This pilot study employed a self-designed pre- and post-survey format to evaluate the effectiveness of virtual “coffee chat” interventions on the knowledge and confidence of ECEs in managing common childhood health issues. Specific health issues that were addressed were diarrhea, cough, trauma, fever, rash, and obesity. These interventions were conducted via individual sessions during the first year of the study. These sessions consisted of a smaller, local cohort with a total of 17 session registrations. The final two sessions, addressing self-regulation and temper tantrums and social and separation anxiety, were conducted the following year and consisted of a larger, statewide cohort of 13 total session registrations. The interventions consisted of an educational session delivered via Zoom, followed by an interactive discussion facilitated by two pediatricians and two medical students. This design enabled us to measure changes in educators’ self-reported knowledge and confidence levels before and after the intervention.

Participants

The study recruited ECEs primarily working in the New River Valley region of Virginia, USA, facilitated through a partnership with the Early Childhood Education Initiatives organization. A designated point person from this organization coordinated recruitment by disseminating session invitations and information to ECEs in the region via multiple email campaigns. For the final two sessions, focusing on “self-regulation and temper tantrums” and “social and separation anxiety”, the invitation was extended statewide through the organization’s connection with a state-level child care organization. This broader outreach allowed child care directors and educators from across Virginia to participate. Participants volunteered based on their availability and interest in learning more about children's health issues. Inclusion criteria were limited to active educators responsible for the care of children in a formal childcare setting. Participation in the study was voluntary, and informed consent was obtained prior to the intervention. While 30 total participant registrations were recorded across the sessions, some educators attended multiple sessions, reflecting ongoing interest and engagement in the intervention. Therefore, the number of unique participants was less than 30.

Needs assessment

Prior to the intervention, a needs assessment was conducted to better understand the specific educational needs and preferences of the participants. This assessment consisted of a survey that collected information on the topics educators were most interested in learning about, as well as demographic information, including the age range of children they worked with, their highest level of education, relevant credentials, and years of experience as an ECE. The survey also included questions about their satisfaction with their current work situation, the biggest challenges they faced in their profession, and the career development resources available to them through their workplace (see Appendix A).

The initial needs assessment was conducted early in the project and received six responses from educators in the New River Valley region. This survey was designed to gather preliminary insights into participant interests, professional challenges, and preferred educational topics. As the project progressed, a "Spring Topic Survey" was sent to the local cohort to refine session topics for future interventions. At the time, the decision to expand the final sessions to a statewide cohort had not yet been made and as a result the follow-up survey did not include participants from the broader group. Despite this limitation, the Spring Topic Survey ensured that the local cohort's priorities continued to shape the intervention’s content (see Appendix B).

Pre- and post-surveys

The effectiveness of the virtual intervention was evaluated using self-designed pre- and post-session surveys administered to participants (see Appendix C for a sample pre-survey). These surveys measured the participants’ attitudes and confidence in managing childhood health concerns, particularly focused on the session’s topic (e.g., self-regulation and temper tantrums). The pre-survey contained questions designed to gauge initial levels of comfort and preparedness in addressing health issues such as:

“I understand what self-regulation is.” “I understand what temper tantrums are.” “I understand what factors can contribute to temper tantrums.” “I currently promote healthy self-regulation habits among children.” “I effectively encourage proper coping skills in children.“ “I find it difficult to advise parents about self-regulation and temper tantrums.” “I understand the implications of poor self-regulation.” “I am aware of resources and strategies available to assist children struggling with self-regulation/temper tantrums and their families.” “There is a need for more education or resources on childhood self-regulation.” “I feel comfortable reaching out to a pediatrician for information.”

Similar questions were asked in the pre-surveys of the other topics. After the educational session, the same survey was administered to evaluate changes in attitudes, confidence, and knowledge (see Appendix D for a sample post-survey). Both surveys included a combination of Likert-scale questions from 1 (strongly disagree) to 5 (strongly agree) and open-ended responses designed to measure the educators’ self-reported knowledge, confidence in managing childhood health issues, and satisfaction with the session. The participants were also asked to rate the value of the session, their likelihood of recommending it to colleagues, and to provide feedback for improving future sessions.

Participants in this study remained anonymous to ensure confidentiality. To facilitate pairing of pre- and post-surveys for analysis while maintaining anonymity, each participant was instructed to create a unique four-digit code using their birth month and date. For example, a participant born on September 12 would enter "0912" as their code. This approach allowed us to link their responses before and after the intervention while safeguarding their identity.

The surveys were administered electronically, and participants were informed about the process of creating and using the code at the time of enrollment. Data collected from the surveys were stored securely, and all responses were analyzed in aggregate form to protect participant privacy.

Educational intervention

Each virtual session was conducted via Zoom and included two pediatricians and two medical students as facilitators. The sessions were structured to address the specific topics identified in the needs assessment. Each session began with a 15-minute presentation tailored to the selected health topic. The presentations were designed to be accessible and informative, providing evidence-based guidelines for identifying and managing common childhood health conditions encountered in daycare and early childhood education settings. Topics were presented in a simplified, practical format that was highly relevant to the daily experiences of ECEs. For example, the presentation on cough covered critical areas, including common causes of cough along with the various possible etiologies such as infection, obstructive lung pathologies, and allergies; differentiating between common and concerning cough symptoms along with red flag symptoms; evidence-based guidelines on when a child with a cough can stay in daycare and when to recommend parental follow-up with a healthcare provider; simple at-home remedies to alleviate discomfort; and prevention strategies. Each presentation followed a similar format, which combined evidence-based guidance, symptom recognition, management strategies, and return-to-care considerations to ensure consistency across topics while tailoring content to each specific health concern.

Following the presentation, an interactive discussion was held, allowing participants to ask questions and clarify specific concerns in real time. This dialogue was intended to deepen the educators’ understanding of the topics and encourage collaboration between healthcare providers and educators. During these discussions, particular emphasis was placed on addressing challenges related to parental collaboration and navigating difficult conversations. Educators were able to share specific scenarios they encountered and facilitators provided practical strategies for these situations, including framing conversations in a positive, supportive manner and using evidence-based information to guide discussions with families. The content of each session was developed in response to the needs identified by the educators themselves, ensuring that the material was not only evidence-based but also directly applicable to the challenges they face in their professional roles.

All sessions were developed collaboratively by the research team, including two board-certified pediatricians and two medical students. The pediatricians brought clinical expertise in pediatrics and child development, while the medical students contributed to session design. Prior to each session, the team held internal planning meetings to discuss the format, review the content, and ensure clarity and alignment with the learning needs of ECEs. Although no formal facilitator training module was developed, these planning sessions served as preparation to ensure consistent delivery, accuracy, and appropriateness of the content across all sessions.

Data analysis

Demographic and needs assessment data were summarized descriptively to provide an overview of participants’ backgrounds, professional challenges, and areas of interest. These findings were used to tailor the intervention content to align with educators' priorities and needs.

Quantitative data collected from pre- and post-surveys were analyzed to assess changes in participants’ knowledge and confidence levels related to managing childhood health concerns. Likert-scale responses were scored numerically (1=strongly disagree to 5=strongly agree) and aggregated to generate total scores for each participant. Paired pre- and post-survey responses were compared to evaluate the intervention's impact. Descriptive statistics, including means and standard deviations, were calculated for each session's pre- and post-survey scores. A Wilcoxon Signed Rank test, a non-parametric method, was performed to assess the statistical significance of changes in scores across sessions, given the small sample sizes and non-normal distribution of the data. Statistical significance was set at p <0.05.

Qualitative data from open-ended survey responses were analyzed to identify common trends and feedback regarding the intervention’s content and delivery. Recurring themes were categorized to provide context for the quantitative findings.

## Results

Needs assessment

The needs assessment was conducted early in the project, with six responses from ECEs in the New River Valley region. Despite the small sample size, the survey provided valuable insights into participant demographics, professional challenges, and educational interests:

Demographics of Survey Participants

Most respondents reported working with children aged two to four years, though several also cared for infants and school-aged children up to six years. Educational backgrounds varied among participants: 50% held associate’s degrees, 33% held bachelor’s degrees, and one participant held a master’s degree (17%). In terms of professional experience, the majority of educators had been in the field for over 15 years, while others reported having between 10-15 years or 5-10 years of experience, each representing 17% of the sample.

Topics of Interest

When asked which topics they were most interested in learning about, participants cited a range of common childhood illnesses, including hand-foot-mouth disease, cough, rash, lice, and fevers. Infection prevention emerged as another key area of interest, specifically regarding how to reduce illness transmission in group-care settings. Additionally, participants expressed a desire for more guidance on communicating with parents about illness management protocols.

Professional Challenges

Educators shared several ongoing challenges they face in their roles. These included difficulties managing time and workload while addressing the individual needs of children, and challenges related to collaborating effectively with parents on health-related matters. Another concern raised was the limited availability of professional development opportunities that are relevant and accessible, making it harder for educators to stay current with best practices in early childhood care.

Satisfaction with Current Role

Respondents rated their job satisfaction with an average score of 3.8/5, indicating moderate satisfaction. Comments suggested a desire for additional support and resources to enhance their roles and improve outcomes for children.

Implications for Intervention Design

The needs assessment results were instrumental in shaping the content of the intervention. By focusing on the specific health topics and professional challenges identified, the sessions ensured relevance and practical application for participants. Although limited to the local cohort, the survey findings laid the foundation for a responsive and adaptable intervention model.

Spring topic survey

To refine the focus of future intervention sessions, a “Spring Topic Survey" was distributed via email to the local cohort of ECEs. The survey was used to identify additional areas of interest and professional challenges that were not addressed in the initial needs assessment. Specific topic requests included: rash, conflict resolutions, and having difficult conversations with children and their families.

Participant demographics

Across all sessions, there were 30 total registrations, but some participants attended multiple sessions. The actual number of unique participants was 16, reflecting educators who engaged in one or more sessions during the study period. This overlap highlights the value participants placed on the content, with some returning for additional topics. Sample sizes varied across individual topics (Table 1). Most participants were from the New River Valley region, with additional statewide representation for the final two sessions on "self-regulation and temper tantrums" and "social and separation anxiety." Participant backgrounds included varying levels of professional experience and educational qualifications, reflecting a diverse group of educators engaged in formal childcare settings.

**Table 1:** Topics showing notable improvements

Topic	Difference
Cough	Pre-score mean of 20.25 ± 1.71 increased to 22.50 ± 2.38, showing a difference of 2.25 ± 1.26.
Falls, Bumps, and Bruises	Pre-score mean of 17.67 ± 0.57 rose to 20.67 ± 1.55, with a difference of 3.00 ± 1.73.
Rash	Pre-score mean of 18.93 ± 5.13 rose to 21.67 ± 2.08, with a difference of 3.33 ± 3.51.
Self-Regulation and Temper Tantrums	The largest cohort (N=10) had pre- and post-scores of 39.10 ± 2.02 and 41.90 ± 4.09, respectively, yielding a difference of 2.80 ± 3.58.

Pre- and post-intervention analysis

The effectiveness of the virtual “coffee chat” sessions was evaluated using pre- and post-surveys, which measured changes in educators' knowledge and confidence in managing common childhood health issues. The analysis included both descriptive statistics and hypothesis testing for overall change scores.

Descriptive statistics by topic

For six of the eight presentations, total scores increased, reflecting overall improvements in self-reported knowledge and confidence levels. Notable improvements were observed in multiple topics, as outlined in Table 1.

**Figure 1:**
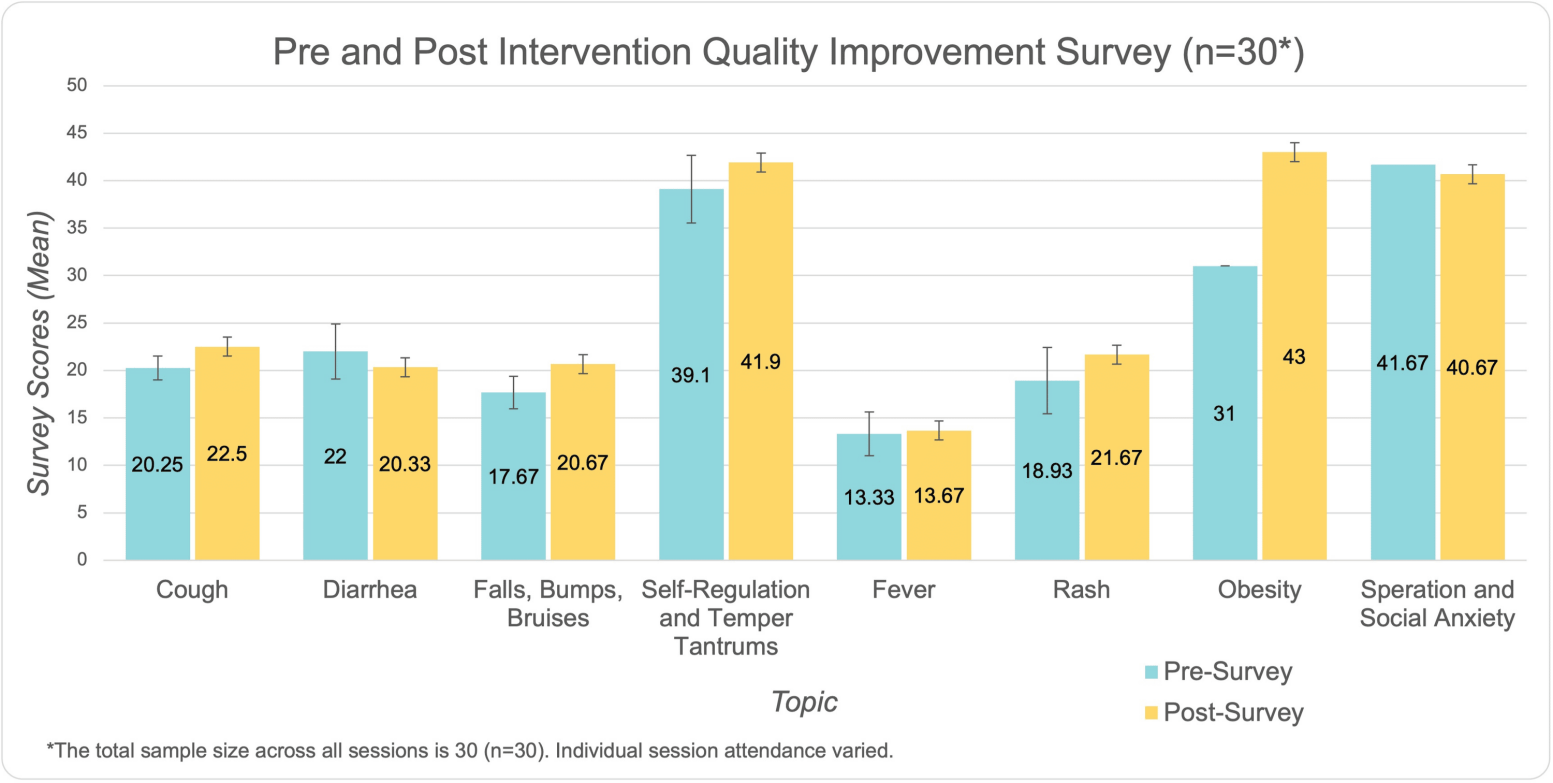
Mean pre-survey and post-survey scores for each topic addressed during the virtual "coffee chat" sessions, illustrating changes in ECEs’ self-reported knowledge and confidence. Mean scores and standard deviation error bars (± values) are displayed for each topic. Pre-survey scores are represented in turquoise, while post-survey scores are depicted in yellow. The p-value was significant at p <0.001.

However, diarrhea and social and separation anxiety showed slight decreases in post-intervention scores, with mean changes of -1.67 ± 2.89 and -1.00 ± 5.00, respectively. These decreases may reflect variability in participant interpretation, session delivery, or differences in how participants responded to the content. Table 2 summarizes the mean scores and standard deviations for pre- and post-survey total scores across all topics. Figure 1 provides a visual comparison of pre- and post-survey scores across all topics, highlighting notable improvements in the areas listed above. While the overall trend supports the effectiveness of the intervention, the slight declines in these two sessions may warrant further exploration or refinement of content delivery in future sessions.

**Table 2:** Mean total scores and standard deviations for pre- and post-surveys across all presentation topics. The data highlights changes in ECEs’ self-reported knowledge and confidence following the virtual "coffee chat" sessions. ECE: Early childhood educator

Presentation	N	Pre	Post	Difference
Cough	4	20.25 ± 1.71	22.50 ± 2.38	2.25 ± 1.26
Diarrhea	3	22.00 ± 1.73	20.33 ± 1.15	-1.67 ± 2.89
Falls, Bumps, Bruises	3	17.67 ± 0.57	20.67 ± 1.55	3.00 ± 1.73
Self-Regulation and Temper Tantrums	10	39.10 ± 2.02	41.90 ± 4.09	2.80 ± 3.58
Fever	3	13.33 ± 1.53	13.67 ± 1.53	0.33 ± 2.31
Rash	3	18.93 ± 5.13	21.67 ± 2.08	3.33 ± 3.51
Obesity	1	31.00 ± 0.00	43.00 ± 0	12.00 ± 0.00
Separation and Social Anxiety	3	41.67 ± 1.15	40.67 ± 5.13	-1.00 ± 5.00

Overall effectiveness

Given the small sample sizes for individual sessions, hypothesis testing was performed on pooled data across all sessions (N=30). A Wilcoxon Signed Rank test revealed a statistically significant overall improvement in post-intervention scores compared to pre-intervention scores (p <0.001), indicating the intervention's success in enhancing educator knowledge and confidence. However, due to the lack of a control group and short-term follow-up, these findings should be viewed as exploratory rather than conclusive evidence of causality. For the session on self-regulation and temper tantrums, a specific test of significance confirmed meaningful improvement (p=0.0041), highlighting the value of this topic for statewide participants.

Participant feedback

Qualitative data from the open-ended responses in the surveys were analyzed thematically to identify common feedback related to the content and delivery of the intervention. Several recurring themes were observed. Many participants expressed that they appreciated the opportunity to engage directly with healthcare professionals and found the information presented to be highly relevant to their daily responsibilities. The practical, evidence-based approach used to address common childhood health concerns was noted as especially beneficial. In addition, participants consistently emphasized the value of the interactive discussion component that followed each presentation. They reported that having the chance to ask questions and explore real-life scenarios enhanced their understanding and made the sessions more impactful.

Session value and recommendations

Regarding the value of the sessions, educators overwhelmingly expressed that the sessions were highly beneficial, with an average score of 4.57/5 on a Likert scale. When asked if they would recommend the session to others, the majority indicated a strong likelihood of recommending it, with an average score of 4.7/5. Educators particularly appreciated the real-time interaction with healthcare professionals, which they felt enhanced their learning experience.

## Discussion

This pilot study aimed to enhance the knowledge and confidence of ECEs in managing common childhood health issues through virtual “coffee chat” sessions. The intervention focused on providing evidence-based information on various health concerns while fostering an interactive dialogue between educators and healthcare professionals. The findings demonstrate that the intervention effectively improved educators' knowledge and confidence, as reflected in the significant positive changes in pre- and post-survey scores across multiple health topics.

Key findings

The results of this study highlight the importance of accessible, practical training for ECEs on managing common health issues. Statistically significant improvements in educators' knowledge and confidence were observed for most topics. For instance, participants reported feeling more prepared to handle issues like coughs and bumps/bruises, with notable increases in their ability to determine when children should stay home or when to seek further medical advice. The self-regulation and temper tantrums session, which was extended statewide, showed meaningful improvement in participants' understanding and confidence in managing behavioral challenges. However, not all sessions showed uniform improvement. The diarrhea session displayed a slight decrease in post-intervention scores, which could suggest variability in educator interpretation or the need for further clarity on the topic. Similarly, the session on social and separation anxiety demonstrated a minor decline in post-session scores, potentially reflecting either increased awareness of the complexity of the issue or differences in how educators interpreted the survey questions. These findings highlight the importance of refining educational content to better meet the diverse needs of educators and ensure that the material resonates with their real-world experiences. Future sessions may benefit from clearer framing of survey items and additional emphasis on practical strategies for emotionally nuanced topics.

Parental collaboration and communication

Although no session was dedicated specifically to parental collaboration or difficult conversations, these topics were addressed informally during the interactive discussions following the presentations. Many educators shared challenges they face when communicating with parents about sensitive issues such as nutrition, hygiene, and illness management. In response, facilitators provided practical advice on how to approach these difficult conversations in a positive, supportive manner, reinforcing the importance of using evidence-based information to guide these discussions. This reflects the educators' expressed interest, as seen in the Spring Topic Survey, in improving communication skills and handling sensitive conversations more effectively.

Strengths and limitations

The study’s strengths include its direct engagement with ECEs and the practical, evidence-based content delivered in an accessible, virtual format. The “Plan, Do, Study, Act” (PDSA) cycle was used to continuously analyze results and properly identify areas of improvement. The iterative feedback from participants through the needs assessment and Spring Topic Survey allowed the intervention to be responsive to educator needs, ensuring the sessions were relevant and timely.

However, the study also had several limitations. The small sample size for each session may limit the generalizability of the results. While the decision to extend the final sessions statewide was an important step, it was not accompanied by a follow-up needs assessment from the larger cohort, which could have provided additional insights into their specific concerns. The overlap in session attendance by some participants further complicates the ability to assess the long-term impact of the intervention on new learners versus those with repeated exposure.

While this pilot study demonstrated improvements in educators’ self-reported knowledge and confidence, it did not assess whether these gains were sustained over time or translated into behavioral changes in classroom practices. Future iterations of the project could include a follow-up phase several months post-intervention to evaluate knowledge retention and real-world application. Additionally, long-term studies could explore whether enhanced educator training positively influences child health outcomes, offering a more comprehensive measure of the intervention’s impact.

Some survey questions, such as “I fear that coughing leads to more serious symptoms/conditions,” may reflect changes in attitudes rather than confidence. For these types of questions, a decrease in scores is consistent with the goal of reducing unnecessary fear, highlighting the intervention's positive impact. However, this contrast with confidence-related questions, where scores are expected to increase, highlights the importance of considering question directionality when interpreting results. Despite this distinction, the overall trends in knowledge and confidence metrics strongly support the intervention's effectiveness. Additional considerations include the lack of formal psychometric validation for the self-designed survey instruments and the absence of a control group. Additionally, anonymity measures were implemented to reduce social desirability bias. As a result, these findings offer meaningful insights that can guide larger, more controlled studies in the future.

Implications for future research and practice

The findings suggest that virtual interventions, like the “coffee chat” sessions, can be an effective model for providing ongoing education and support to ECEs. Future research could focus on a larger, more diverse cohort and incorporate more frequent follow-ups to measure long-term retention and application of knowledge. Additionally, refining the educational content based on participant feedback would ensure that future sessions better meet the needs of educators.

Novelty and importance of the intervention

While there have been other interventions aimed at improving the knowledge and preparedness of childcare staff in managing health concerns, few studies have specifically targeted a wide range of common childhood health issues in a format that is both interactive and accessible [1,14-16]. Our intervention stands out by offering a flexible, virtual platform where educators can receive real-time expert guidance, ask questions, and engage in discussions tailored to the specific health challenges they face.

This format also addresses a key barrier identified in the literature: the limited availability of in-person training [13]. Virtual sessions, as demonstrated in our project, can reach a wider audience, reduce time constraints, and provide ongoing support for educators. The positive feedback from participants highlights the value of this approach, with many expressing a desire for continued sessions and recommending the training to others. This reinforces the feasibility and effectiveness of using virtual platforms for professional development in early childhood education.

## Conclusions

This pilot study showed that virtual “coffee chat” sessions could significantly improve ECEs' knowledge and confidence in managing common health and behavioral concerns. The sessions provided practical, evidence-based strategies tailored to the specific needs of educators, making them both relevant and applicable to childcare settings. The results highlight the effectiveness of this intervention in fostering educator preparedness and addressing gaps in their training, particularly in regions with limited access to professional development resources. While most topics showed strong improvements, the slight decline in post-survey scores for the diarrhea and social and separation anxiety sessions suggests areas where further clarification or adjustments may be beneficial.

The study's findings suggest that virtual sessions are not only feasible but also beneficial for educators managing demanding schedules. Participants’ feedback emphasized the importance of the interactive format, which allowed them to address real-life challenges and strengthen their collaboration with healthcare professionals. Future research should aim to expand the intervention to larger, more diverse cohorts and focus on refining content to ensure it addresses the varied experiences of educators. With continued development, virtual training models like this can play a critical role in improving the quality of care in early childhood education and the health outcomes of children in these settings.

## Appendices

Appendix 1 

**Table 3:** Needs assessment

Question	Response Type	Options (if applicable)
Please enter your personal identifying number. It is your birth month followed by your birth date. For example, someone born on September 12th would enter 0912.	Short Answer	—
If you had the option to receive further information on medical topics pertaining to your profession, what topic(s) would you choose?	Open-Ended	—
What age groups of children do you work with?	Multiple Choice (check all that apply)	Infants (0–12 months), Toddlers (1–3 years), Preschool (3–5 years), School-aged children (6 years and above), Other (please specify): ______
What is your highest level of education?	Multiple Choice	High school diploma, Associate’s degree, Bachelor’s degree, Master’s degree, Other (please specify): ______
Do you have any credentials/license? If so, which one(s)?	Open-Ended	—
How many years of experience do you have as an early childhood educator?	Multiple Choice	Less than 1 year, 1–5 years, 5–10 years, 10–15 years, More than 15 years
How satisfied are you with your current working situation?	Likert Scale	1=Very Dissatisfied to 5=Very Satisfied
What keeps you in your role/child care education field?	Open-Ended	—
What is the biggest challenge you face in your profession?	Open-Ended	—
Are there opportunities for advancement in your career?	Open-Ended	—
If you have a preference of working with a certain age group (i.e. infant, toddler, etc), does your workplace allow you to do so?	Open-Ended	—
What career and development resources are you aware of that are available to you through your workplace?	Open-Ended	—

Appendix 2 

**Table 4:** Spring Topic Survey

Question	Response Type	Options (if applicable)
In order to ensure your privacy, the information you enter will be associated only to a 4-digit personal identifying number rather than your name or email. Your identifying number is your birth month followed by your birth date. For example, someone born on September 12th would enter 0912.	Short Answer	—
Have you previously completed the demographic questions (for example, "What is your highest level of education?")? If you have, please select "Yes" to proceed directly to the Topic Survey. If you have not or are not sure, please select "No" and complete the demographic questions.	Multiple Choice	- If “Yes”, then participants would continue filling out the following survey. - If “No” was selected, then the participant would be directed to the “Needs Assessment” survey seen in Appendix A, followed by the questions listed below.
What medical topics pertaining to your profession would you like to receive further information on? If you have other recommendations, please write them in the "Other" option.	Multiple Choice (check all that apply)	Rash, Trauma (falls, injuries, etc.), Poisoning, Gastrointestinal issues (diarrhea, vomiting, etc.), Seizures, Conflict resolutions (parental communications, expectations, etc.), Runny/snotty nose, Having difficult conversations (brushing teeth, nutrition, etc.), Other (please specify): ______
If you selected “Conflict resolutions,” please share any situations or cases that you would like to discuss/have us cover.	Open-Ended	—
If you have any questions, comments, or concerns, please list them here as your feedback is valuable to us.	Open-Ended	—

Appendix 3 

**Table 5:** Pre-survey example (Cough)

Question	Response Type	Options (if applicable)
I feel scared when a child has a cough.	Likert Scale	1=Strongly Disagree — 5=Strongly Agree
I feel well prepared to manage a child with a cough.	Likert Scale	1=Strongly Disagree — 5=Strongly Agree
I fear that coughing leads to more serious symptoms/conditions.	Likert Scale	1=Strongly Disagree — 5=Strongly Agree
I know when I should administer appropriate medications for a cough.	Likert Scale	1=Strongly Disagree — 5=Strongly Agree
I feel confident advising parents about their child’s cough.	Likert Scale	1=Strongly Disagree — 5=Strongly Agree
I can determine whether a child with a cough should remain in childcare.	Likert Scale	1=Strongly Disagree — 5=Strongly Agree
I feel comfortable reaching out to a pediatrician for information.	Likert Scale	1=Strongly Disagree — 5=Strongly Agree
What determines your next step when a child has a cough?	Multiple Choice (check all that apply)	Personal Beliefs, Prior Experiences, Prior Training/Education, Workplace Policy, CDC Guidelines, Other (please specify): ______

Appendix 4 

**Table 6:** Post-survey example (Cough)

Question	Response Type	Options (if applicable)
I feel scared when a child has a cough.	Likert Scale	1=Strongly Disagree — 5=Strongly Agree
I feel well prepared to manage a child with a cough.	Likert Scale	1 = Strongly Disagree — 5=Strongly Agree
I fear that coughing leads to more serious symptoms/conditions.	Likert Scale	1=Strongly Disagree — 5=Strongly Agree
I know when I should administer appropriate medications for a cough.	Likert Scale	1=Strongly Disagree — 5=Strongly Agree
I feel confident advising parents about their child’s cough.	Likert Scale	1=Strongly Disagree — 5=Strongly Agree
I can determine whether a child with a cough should remain in childcare.	Likert Scale	1=Strongly Disagree — 5=Strongly Agree
